# Predictivity Approach for Quantitative Structure-Property Models. Application for Blood-Brain Barrier Permeation of Diverse Drug-Like Compounds

**DOI:** 10.3390/ijms12074348

**Published:** 2011-07-05

**Authors:** Sorana D. Bolboacă, Lorentz Jäntschi

**Affiliations:** 1 “Iuliu Haţieganu” University of Medicine and Pharmacy Cluj-Napoca, 13 Emil Isac, 400023 Cluj, Romania; E-Mail: sbolboaca@umfcluj.ro; 2 University of Agricultural Sciences and Veterinary Medicine Cluj-Napoca, 3-5 Calea Mănăştur, 400372 Cluj, Romania

**Keywords:** *in silico* prediction, partition-coefficient, blood-brain barrier (BBB), permeation, structure-property relationship (SPR), molecular descriptors family on vertices cutting (MDFV)

## Abstract

The goal of the present research was to present a predictivity statistical approach applied on structure-based prediction models. The approach was applied to the domain of blood-brain barrier (BBB) permeation of diverse drug-like compounds. For this purpose, 15 statistical parameters and associated 95% confidence intervals computed on a 2 × 2 contingency table were defined as measures of predictivity for binary quantitative structure-property models. The predictivity approach was applied on a set of compounds comprised of 437 diverse molecules, 122 with measured BBB permeability and 315 classified as active or inactive. A training set of 81 compounds (~2/3 of 122 compounds assigned randomly) was used to identify the model and a test set of 41 compounds was used as the internal validation set. The molecular descriptor family on vertices cutting was the computation tool used to generate and calculate structural descriptors for all compounds. The identified model was assessed using the predictivity approach and compared to one model previously reported. The best-identified classification model proved to have an accuracy of 69% in the training set (95%CI [58.53–78.37]) and of 73% in the test set (95%CI [58.32–84.77]). The predictive accuracy obtained on the external set proved to be of 73% (95%CI [67.58–77.39]). The classification model proved to have better abilities in the classification of inactive compounds (specificity of ~74% [59.20–85.15]) compared to abilities in the classification of active compounds (sensitivity of ~64% [48.47–77.70]) in the training and external sets. The overall accuracy of the previously reported model seems not to be statistically significantly better compared to the identified model (~81% [71.45–87.80] in the training set, ~93% [78.12–98.17] in the test set and ~79% [70.19–86.58] in the external set). In conclusion, our predictivity approach allowed us to characterize the model obtained on the investigated set of compounds as well as compare it with a previously reported model. According to the obtained results, the reported model should be chosen if a correct classification of inactive compounds is desired and the previously reported model should be chosen if a correct classification of active compounds is most wanted.

## 1. Introduction

The blood-brain barrier (BBB), complex membranous system of brain capillary endothelial cells, pericytes, astrocytes, and nerve endings, plays an essential role in maintaining the homeostasis of the central nervous system by blocking the movement of molecules [1]. Determination of blood-brain barrier penetration is crucial in the assessment of compounds suitability as central nervous system drug [2]. As the population’s life expectancy increases and neurological pathologies become more frequent, there is a need to rapidly and cost and resource effectively identify potentially adverse effects of drugs acting as CNS (central nervous system) and non-CNS targets [3,4].

Quantitative structure-activity/property relationship models support the “fail fast, fail cheap” model [5] in analysis of the link between structure of the compounds and associated activity/property. Different techniques have been used in BBB modeling. One of the earliest predictions of BBB permeation was the one presented by Young *et al*. [6] as a linear relationship between logBB and ΔlogP (histamine H_2_ receptor agonists).

Crivori *et al*. used descriptors from 3D molecular fields to estimate the BBB and identified a model able to correctly predict 90% of the permeation data [7]. Narayanan and Gunturi used a systematic variable selection and modeling method based on the prediction on a sample of 88 BBB compounds and identified as best performing one model with three descriptors and one model with six descriptors with higher performances [8]. These models proved to have a success ratio of 82% in predicting the BBB + external data set. Statistical characteristics of their best models are presented in Equation 1 and Equation 2.

(1) $$R=0.8425,R_{\text{loo}}=0.8239,F=68.49,se=0.4165,j=3,n=88$$

(2) $$R=0.8638,R_{\text{loo}}=0.8472,F=60.982,se=0.3919,j=6,n=88$$

where *R* = correlation coefficient, *R*_loo_ = leave-one-out correlation coefficient, *F* = *F*-value, *se* = standard error of estimate, *j* = number of descriptors in the model, *n* = sample size.

Subramanian and Kitchen [9] identified that logP, polar surface area and some electrotopological indices are able to provide accurate predictive model for logBB (logarithm of brain to plasma concentration ratio). Linear regression and multivariate genetic partial least squares approaches were applied and the obtained model proved to have a success rate higher than 70% for active compounds and almost 60% for inactive compounds [9]. Subramanian and Kitchen concluded that the prediction consensus was not able to significantly improve the discrimination of active and inactive molecules on central nervous system.

Goodwin and Clark analyzed and presented the main problems of *in silico* prediction of blood-brain barrier penetration: quality of measured data available and prediction uncertainty and relevance of predictive models [10]. They pointed out the usefulness of local and global models as well as of the accuracy of experimental data by highlighting some success stories [11–13].

Non-linear approaches have also been used to predict the distribution of compounds based on different states (neutral, cationic and anionic) of the compounds distributed into three different compositions (lipid, protein and water) [14]. The statistical characteristics of the predictive model for the distribution of compounds were as follows [14]: All data: *R* (correlation coefficient) = 0.906, *se* (standard error) = 0.326, *n* (sample size) = 160; Training set: *R* = 0.908, *se* = 0.320, *n* = 139; and Test set: *R* = 0.903, *se* = 0.297, *n* = 21.

Klon reviewed the computational models of central nervous system penetration according to the type of variable of interest (quantitative–for logBB models and qualitative–for binary models) [15]. He proposed the permeability surface product and the fraction unbound in the brain as appropriate metric endpoints [15]. Although, due to the availability of the experimental data, the blood:brain ratio is still used for *in silico* modeling [16,17].

Nowadays, many models for prediction of logBB are available in the literature. However, how the best model could be identified? How can different models be compared to one another? A new classification model based on multi-linear regression to the domain of blood-brain barrier modeling is introduced in this manuscript. A series of 15 statistical parameters were introduced to be used as diagnostic tool of a binary logBB model as well as for the comparison of different classification models. The study aimed to present a new approach in assessment of a predictivity of a structure-based prediction model and was effectively accomplished.

## 2. Results

The multiple linear regression (MLR) that accomplished as many criteria as possible and proved to perform best is presented in Equation 3 (a—MLR equation, b—statistical characteristics of MLR model, c—statistical characteristics model in leave-one-out analysis).

(3a) $$\begin{array}{c} {\hat{Y}}_{\text{logBB}}=0.5370(\pm 0.30)-8.4411(\pm 4.42)\times \text{TLgFAIDI}-497.0205(\pm 144.97)\times \text{GAmIAaDI}+\\ 4.1129(\pm 1.55)\times \text{TAgFIADL}-3.1303(\pm 1.26)\times \text{TAgPIADL} \end{array}$$

(3b) $$\begin{array}{c} R=0.7816\;(95\%{\text{CI}}_{\text{r}}\;[0.6791-0.8541]),R^{2}=0.6109;\\ se_{\text{est}}=0.61;n_{\text{tr}}=81;F_{\text{est}}(p)=30\;(6.41\times 10^{-15})\\ t_{\text{X}1}(p)=3.59\;(5.84\times 10^{-4});{\text{t}}_{\text{X}2}(p)=-3.80\;(2.87\times 10^{-4});t_{\text{X}2}(\text{p})=-6.83\;(1.85\times 10^{-9});\\ t_{\text{X}4}(p)=5.30\;(1.11\times 10^{-6});t_{\text{X}5}(p)=-4.96\;(4.21\times 10^{-6}) \end{array}$$

(3c) $$\begin{array}{c} R_{\text{loo}}=0.7334;R^{2}{}_{\text{loo}}=0.5378;s_{\text{loo}}=0.65;F_{\text{loo}}(p)=22\;(4.27\times 10^{-12});\\ R\;(p)=0.7816\;(7.31\times 10^{-18});{\text{r}}_{\text{sQ}}(p)=0.7636\;(9.18\times 10^{-17});\\ \rho (p)=0.7460\;(8.91\times 10^{-16});\tau_{\text{a}}(p)=0.5568\;(1.37\times 10^{-10});\tau_{\text{b}}(p)=0.5578\;(1.53\times 10^{-10});\\ \tau_{\text{c}}(p)=0.5499\;(2.16\times 10^{-10});\Gamma (p)=0.5589\;(8.86\times 10^{-5}) \end{array}$$

where &*Ycirc;*_logBB_ = property estimated by MDFV model; TLgFAIDI (*X*_1_), GAmIAaDI (*X*_2_), TAgFIADL (*X*_3_), and TAgPIADL (*X*_4_) = members of MDFV; the values in round brackets allow us to obtain the lower (subtraction) and upper (addition) confidence boundary for the slope parameters; *R* = correlation coefficient; *R*^2^ = determination coefficient; *s*_est_ = standard error of estimate; *n*_tr_ = sample size-training set; *F*_est_ (p) = *F*-value of the model (*p*-value); *t* = *t*-value; *R*^2^ _loo_ = cross-validation leave-one-out square correlation coefficient; *s*_loo_ = standard error of predicted; *F*_loo_ = *F*-value on cross-validation leave-one-out model; values in the [] = 95% confidence interval; *r* = Pearson correlation coefficient between property observed and estimated by the model; *r*_sQ_ = semi-quantitative correlation coefficient; *ρ* = Spearman rank correlation coefficient; *τ*_a_, *τ*_b_, *τ*_c_ = Kendall’s correlation coefficients; *Γ* = Gamma correlation coefficient. The descriptor’s contributions to the logBB of investigated compounds are as follows:

▪ Interaction Via: bonds (topology – **T**LgFAIDI, **T**AgFIADL, and **T**AgPIADL) & space (geometry – **G**AmIAaDI);
▪ Dominant Atomic Property: electronic affinity (G**A**mIAaDI, T**A**gFIADL, and T**A**gPIADL) & melting point (T**L**gFAIDI);
▪ Interaction Descriptor: related to inverse of property and distance (TA**g**FIADL, TA**g**PIADL GA**m**IAaDI);
▪ Structure on Property Scale: logarithm (TAgFIAD**L** and TAgPIAD**L**) & identity (TLgFAID**I** and GAmIAaD**I**).

Two descriptors (TAgFIADL and TAgPIADL) proved to correlate significantly (a perfect concordance between all seven correlation methods) but neither of them significantly correlates with the observed property. No other statistically significant correlations could be identified between descriptors or between descriptors and logBB. The Durbin-Watson statistics was computed as a measure of autocorrelation. A value of 2.108 was obtained for the model presented in Equation 3.

A concordance of 69% (also known as accuracy) was obtained for training set after transformation of observed and estimated logBB as dichotomial variables. The concordance according with classification of compounds as active and inactive (based on observed value) are known as sensitivity and specificity.

The prediction ability of the model presented in Equation 3 was investigated on the test set. The obtained statistical characteristics are presented in Equation 4.

(4) $$\begin{array}{c} R=0.7060\;(95\%\text{CI }[0.5088-0.8327]);R^{2}=0.4985;\\ se_{\text{pred}}=0.76;n_{\text{ts}}=41;F_{\text{pred}}(p)=9\;(2.92\times 10^{-5})\\ r(p)=0.7060\;(1.55\times 10^{-7})\;r_{\text{sQ}}(p)=0.7459\;(1.78\times 10^{-8});\rho (p)=0.7787\;(1.27\times 10^{-9});\\ \tau_{\text{a}}(p)=0.5780\;(3.94\times 10^{-6})\;\tau_{\text{b}}(p)=0.5816\;(4.43\times 10^{-6});\tau_{\text{c}}(p)=0.5640\;(6.29\times 10^{-6});\\ \Gamma (p)=0.5852\;(3.01\times 10^{-3}) \end{array}$$

where *se*_pred_ = standard error of predicted; *n*_ts_ = sample size of test set; *F*_pred_ = *F*-value of predicted.

The concordance between observed and predicted property when classification was applied on the test set proved to be of 73% (accuracy, see Table 1). The ability of the classification model, which proved not to be a very good model in terms of goodness-of-fit, was analyzed for the training, test and external sets with the defined diagnostic parameters. The results are presented in Table 1.

The comparison of correlation coefficient obtained by the model from Equation 3 with the models from Equation 1 (Steiger’s *Z* test = 1.15, *p* = 0.13) and Equation 2 (Steiger’s *Z* test = 1.65, *p* = 0.05) showed that the classification model is neither better nor worse in terms of goodness-of-fit.

The proposed statistical parameters were applied as diagnostic tools for the model presented in Equation 2 and the results are presented in Table 2.

## 3. Discussion

*In silico* modeling has been revolutionized along with the development and improvement of computers and information technologies [18,19]. Chemoinformatics, bioinformatics, combinatorial chemistry [20], high throughput screening [21], virtual screening, de novo design [22], structure-based drug design [23–25] are approaches frequently used in the processes of drug discovery. The study aimed to present a new approach in the assessment of the predictivity of a structure-based prediction model and was effectively accomplished. A predictive model has been developed based on a family of structural descriptors (molecular descriptors family on vertices cut) using the multiple-linear regression method. The best performing MLR model was identified to accomplish a series of criteria [26] and its performances were assessed using statistical parameters computed on the 2 × 2 contingency table.

The models with the highest correlation coefficient, the highest Fisher parameter, the lowest standard error of estimate, and the smallest possible number of significant parameters was chosen (see Equation 3). All four descriptors used by the model had their significant contribution to the explanation of the BBB permeation, as it can be observed from Equation 3. The analysis of the best performing model in terms of descriptor’s contribution to the property (permeation of blood-brain barrier of drug-like compounds) revealed the following:

▪ almost 61% of the variation of BBB permeability could be explained by the linear-relationship with structural-based descriptors; the interaction between property and structure is performed through bonds (topology) and space (geometry–first letter in descriptor’s name);
▪ the penetrability of drug-like compounds proved to be related to electronic affinity (*A*–second letter from descriptor’s name) and melting point under normal temperature and pressure conditions (*L*) of BBB compounds; the structure on property scale proved to be of identity (*I*–last letter in descriptor’s name), and logarithm (*L*) type.

The obtained model proved to be a reliable model with a reasonable goodness-of-fit since the sample size was so heterogenous. The absence of statistically significant correlation between descriptors and the Durbin-Watson statistics (based on its value, the presence of autocorrelation was withdrawn [27,28]) sustained the reliability of the MLR model. The results obtained in leave-one-out cross validation showed that the model could have abilities in prediction on external data since the difference between determinations was of 0.07 [29]. Based on results obtained in leave-one-out cross validation analysis, we expected the difference between the model obtained in the training set and its performances on the test set not to be higher/smaller than 12% in terms of the determination coefficient. The test set, a set that comprised a number of 41 drug-like compounds with known permeation on blood-brain barrier, was analyzed for its prediction and penetration abilities. As expected, the determination coefficient was small compared to the determination coefficient obtained on the training set but proved not to significantly different since the associated 95% confidence intervals overlap with one another.

The goodness-of-fit of our model (Equation 3) was compared with two previously reported models [8] and proved neither better nor worse in terms of the correlation coefficient. Although, the following should be taken into account and should give weight to the model presented in Equation 3:

- The number of descriptors used by our model is 4 (Equation 3) while the number of descriptors used by best performing previously model is 6 (Equation 2).
- The number of compounds in the training set was almost the same but the compounds included were not identical. It is well known that if some compounds are included or excluded from analysis, similar MLR equations could be obtained but with some changes of parameters. The quality criteria used to determine if a compound would be included in the sample were as follows:
  ▪ reliable experimental data (the compounds with different values of experimental data obtained by applying the same protocol were not included);
  ▪ compound identity (one compound was included whenever identical compounds were identified);
  ▪ normality of experimental data.

Furthermore, the abilities of the obtained model (Equation 3) to classify correctly the permeation of the blood-brain barrier were tested on two samples of compounds: the test set and the external set (compounds used neither in the training nor in the test sets). This analysis was carried out after transformation of observed blood-brain barrier permeation as a dichotomous variable; the interpretation of the obtained statistical parameters (see Table 1) revealed the following:

▪ The presence of dependence between classification and observed permeation obtained for all three sets of compounds showed that the model has abilities in estimation as well as in prediction.
▪ The total fraction of compounds correctly classified proved to be almost identical in the training and test sets. Even if the accuracy of the classification model was smaller in the training set compared to the test and external sets, the accuracies proved not to be significantly different since their confidence intervals overlapped one another.
▪ The error rate (the fraction of compounds misclassified) proved to vary from 27% to 31% with a higher value obtained in the training set compared to the test and external sets.
▪ A valid classification model is the one that is able to classify correctly as many compounds as possible. Thus, it is expected that the 95% confidence interval of prior proportional probability of an active compound to overlap on the confidence interval of post-test probability of classification as active for a good classification model. The prior probability of an active class and the post-test probability of classification (where the test is our classification model) sustain the ability of the model in classification. The smallest difference for the active class of compounds was seen in training set; the same conclusion has also been seen for the inactive class of compounds.
▪ The classification model proved to have higher abilities in the identification of a true active compound out of all the active compounds in the test set. Since the associated confidence intervals associated to sensibility overlap one another in the training and test sets, the model has the same ability to identify the true active compounds in these sets. Analyzing the sensitivity of the classification model on external set showed that it is not appropriate to use this classification model to classify active BBB permeation compounds since the false negative rate is almost 60% (there is a 2/5 chance to correctly classify an active BBB compound).
▪ The higher ability in the classification of an inactive compound was obtained in an external set (~86%). This ability seems not to be significantly different in the training, test and external sets since the associated 95% confidence intervals overlap one another.
▪ The higher positive predictivity proved to be obtained in the training set and refer to the ability of our classification model to correctly assign a compound as active out of all active assigned compounds. As expected, by analyzing the sensitivity, the positive predictivity of our model was significantly smaller when the classification model was applied to the external set (~56%, but the confidence interval did overlap with the confidence interval of positive predictivity obtained on the training set).
▪ The highest value of negative predictivity was obtained in the test set. The negative predictivity proved not to be significantly different between all three investigated sets.
▪ The smallest value of the probability of wrong classification as an active compound was obtained in the training set while the highest value was obtained in the external set (these two probabilities proved significantly different).
▪ The smallest value of the probability of wrong classification as an inactive compound was obtained in test set. No statistically significant difference in terms of the probability of wrong classification was identified when all three sets of compounds were analyzed.
▪ The odds of correct classification in the group of active compounds divided by the odds of incorrect classification in the group of inactive compounds proved to be almost identical in the training and external sets. Even if the value of the odds ratio obtained in test set is higher than the values obtained in training or external sets, the ability of our classification model in terms of OR proved not to be significantly different in this set (the confidence intervals overlap one another).

The differences in performances of our classification model on the training, test and external sets could be explained by the distribution of active and inactive compounds in the sets (active compounds proved to ~48% in training set, ~46% in test set and ~30% in external set). If the percentage of active compounds in the external set is imposed to be close to the percentage of active compounds in the training and test sets, the parameter’s significant difference will be improved and the classification model could have the same ability in the external set as in the training and test sets.

The same statistical parameters proposed to diagnose the classification model were also computed for the model previously obtained and presented in Equation 2 in order to be used as comparison parameters. These parameters could be used to compare different models obtained on the same but not identical classes of compounds. The comparative analysis of the obtained statistical parameters for the proposed classification model (see Table 1) and for the previously reported model (see Table 2) revealed the following:

▪ The interdependence between the observed and estimated/predicted class (active/inactive) was proved statistically for both models (Equation 3–Table 1 and Equation 2–Table 2) but the correlation coefficients in the 2 × 2 contingency table proved to be higher for the model presented in Equation 2.
▪ The higher accuracy for all three sets sustains the use of model presented in Equation 2 in the classification of active and inactive BBB compounds. The accuracy seems not to be statistically significantly different when the model from Equation 3 is compared to the model from Equation 2 since the associated 95% confidence intervals overlap one another.
▪ The under-classification as well as over-classification seems to have the smallest values for the model from Equation 2 compared to the model from Equation 3, but since the associated 95% confidence intervals overlap one another these differences do not seem to be statistically significant.
▪ The percentage of compounds correctly assigned as active out of all of those assigned as active proved to be higher for the model presented in Equation 2 compared to the model presented in Equation 3. Since the 95% confidence intervals overlap one another, these percentages are not statistically significantly different. The same observation is also true for the percentage of compounds correctly assigned as inactive out of all of those assigned as inactive.
▪ The previously reported model seems to have the smallest probabilities of wrong classification as active/inactive compounds. However, based on the overlap of associated 95% confidence interval it could be that these differences are not statistically significant.

The proposed four descriptors model demonstrates its abilities in the estimation and prediction of BBB drug-like penetration. The “best model” approach could be questionable in terms of goodness-of-fit, but the proposed four descriptors model proved to be good in certain applicability domains as shown above. Moreover, a model with four descriptors may perform worse than a model with six descriptors, but experience may show that the model with four descriptors could be more stable when changing the training data. Consequently, the best idea followed in the paper was to provide a tool to assess the models from certain points of view, and to let the user select their best classifier to fit their chosen applicability domain.

The goodness-of-fit of our model is similar with the goodness-of-fit of other models published in specialty literature when similar sample sizes were used in modeling (*n*_training_ = 329, *R*^2^ _training_ = 0.52, *n*_test_ = 141, *R*^2^ = 0.54, *n*_external_ = 174, *R*^2^ = 0.65 [17]). The classification abilities of a qSAR/qSPR model could be tested using a series of parameters computed based on a 2 × 2 contingency table. The model’s ability to correctly classify BBB drug-like compounds as well as the fraction of compounds misclassified proved not to be significantly different when all three sets were compared. The identified difference in under-classification for the external set, where the false negative rate proved to be significantly higher compared to the values obtained in training and test set could be explained by the different percentage of active compounds in external set compared to training and test sets. Our classification model performs better if the prior proportional probabilities of active and inactive class are closer to each other in investigated sets of compounds (see Table 1). Our model could be applied to classify BBB penetration of drug-like compounds and provide more accurate classification for inactive compounds if the prior proportional probability of an active class is close to the prior probability of an active class obtained in training set. Our classification model could be refined by clustering the observed penetration and obtaining models for each cluster since the training, test and external sets are comprised of drug-like compounds with heterogenous structures.

The presented approach introduced a new concept in the assessment of structure-based drug design: the assessment of the link between the structure of the compound and BBB penetrability through a series of parameters able to show the performances of the model in terms of accuracy, sensitivity, specificity, positive predictivity, *etc*. This approach is relevant to address to the situation when classification as active/inactive compounds is desired. The model with the highest accuracy, sensitivity and specificity must be chosen when more than one qSAR/qSPR model is accessible anytime. The structure that must be investigated is similar to those based on each of the obtained models.

## 4. Experimental Section

### 4.1. Classification Model—Predictivity Approach

The ability of the identified model in the classification of active and inactive BBB compounds (the observed property being greater than or equal to 0 identifies an active compound, otherwise the compound was considered inactive) was assessed using appropriate statistical methods. Concordance defined as identical classification of a compound based on observed and estimated/predicted property was summarized as a percentage and an associated 95% confidence interval.

The performances of the classification model were assessed in training, test, and external sets. The external set is comprised of 315 different drug-like compounds classified as active (71) or inactive (244) BBB compounds. The compounds from the external set were taken from [30] (see Supplementary Material).

The parameters presented in Table 3 were used to assess the classification model. Some parameters were defined by Cooper *et al*. [31] while others were adapted from medical diagnosis studies [32]. The associated confidence intervals under binomial distribution assumption [33,34] were computed for each parameter [35].

### 4.2. Classification Model as Comparison Tool

The proposed statistical approach and associated significance levels were also computed for models presented in Equation 2 in order to be compared with a model introduced in the present manuscript (Equation 3).

Sub-sections 4.3. and 4.4. show how the model presented in Equation 3 was obtained.

The model presented in Equation 3 was compared with previously reported models (Equation 1 and Equation 2) in terms of goodness-of-fit using Steiger’s *Z* test [36] at a significance level of 5%.

### 4.3. Datasets and BBB Permeation Property

A sample of drug-like compounds with blood-brain barrier permeation (known logBB, the blood-brain distribution is expressed as the ration of the steady state molar concentration of a compound in the brain and in the blood) was identified to be included in the analysis [8,37–39]. The quality criteria used to include a compound in the sample were as follows: ▪ reliable experimental data (the compounds with different value of experimental data obtained by applying the same protocol where not included); ▪ compound identity (one compound was included whenever identical compounds were identified); ▪ normality of experimental data.

Two databases were used in order to search the structures of the compounds: PubChem (http://pubchem.ncbi.nlm.nih.gov/, the compound ID is CID followed by a number in Table 4) and ChemSpider (http://www.chemspider.com/, the compound is CSID followed by a number in Table 4). The HyperChem 8.0 was used to draw the compounds that were not identified in the PubChem or in ChemSpider databases.

The compound name, ID (CID-ID of compounds taken from PubChem database or CSID-ID of compounds taken from ChemSpider database) and the observed property expressed in logarithmic scale are presented in Table 4.

The observed property of drug-like compounds included in analysis had a mean of −0.2180 (95%CI [−0.3930; −0.0492]), and a standard deviation of 0.9767. The set presented in Table 4 was randomly split into a training and test set, with ~2/3 of compounds in the training set. The method of randomization was implemented in order to ensure the normal distribution of the observed property in both sets. Descriptive statistics and normality test results for the training and test sets are presented in Table 5.

### 4.4. Molecular Descriptors Calculation

The HyperChem 8.0 was used to optimize the geometry of compounds by using a home-made program [40]. A series of home-made programs were used to perform the following tasks: (1) transform the *.sdf and *.mol files in *.hin files; (2) identify invalid compounds; (3) optimize the geometry of compounds; (4) calculate the molecular descriptors; (5) assign the compounds in training or test sets; (6) select valid descriptors (Jarque-Bera value higher than critical value for the observed activity, identity analysis and inter-correlation analysis); (7) multiple linear regression.

The Molecular Descriptor Family on Vertices approach (MDFV, [41]) was used to calculate the structural descriptors. The calculation of MDFV members is based on candidate fragments obtained using cutting atoms (as vertices cut) on the matrix representation of the molecular graph. A series of home-made PHP programs were developed to compute the MDFV values. The programs are run on an IntraNet network on a FreeBSD server and the results for previously investigated datasets are available online at http://l.academicdirect.org/Chemistry/SARs/MDFV/ (password provided by request). The calculation of descriptors on new data sets of compounds could be made upon request. A total number of 831 descriptors proved to be valid and were used to identify the best performing model.

The model that accomplishes the following criteria was considered the best classification MLR model [42]: highest explanation of the observed logBB (highest correlation coefficient); smallest number of MDFV descriptors; lowest standard error of estimate; highest F-value and smallest associated p-value; smallest difference between correlation coefficient and leave-one-out correlation coefficient, F-value and associated p-value in leave-one-out analysis.

SPSS 16.0 was used to investigate multi-collinearity of descriptors in the MLR (multiple linear regressions) model, auto-correlations and homoscedacity.

## 5. Conclusions

The proposed predictivity approach could be used in the diagnosis of structure-based models (quantitative structure-property relationships or quantitative structure-activity relationships) but also could be seen as a tool for choosing the proper model for the assessment of new compounds. This approach is able to identify the model with the highest ability to identify active or inactive compounds. The best model could be considered the one with highest accuracy, specificity and sensibility as well as the smallest values of false-negative and false-positive rate and smallest values of probability of wrong classification as active or inactive compounds.

In regards to the blood-brain barrier permeation domain, the model presented in this manuscript proved to have high abilities in correct classification of inactive compounds (~86% of inactive compounds from external validation set—315 compounds—were correctly classified as inactive). The previously reported model proved to have high abilities in the correct classification of active compounds (~76% of active compounds from external validation set—92 compounds—were correctly classified as active). Therefore, the reported model should be chosen if the correct classification of inactive compounds is desired and the previously reported model should be chosen if the correct classification of active compounds is most wanted.

## Figures and Tables

**Table 1:** Diagnostic of classification model presented in Equation 3.

Parameter (Abbreviation)	Equation	Training Set (*n* = 81) [95%CI]	Test Set (*n* = 41) [95%CI]	External Set (*n* = 315) [95%CI]

χ^2^ statistic (*p*-value)		10.29 (0.0013)	7.75 (0.0054)	28.24 (*p* < 0.0001)

Φ		0.3564	0.4347	0.2994

Accuracy (AC)	5	69.14 [58.53–78.37]	73.17 [58.32–84.77]	72.70 [67.58–77.39]

Error Rate (ER)	6	30.86	26.83	27.30

Prior proportional probability of	7			
-an active class		0.482 [0.371–0.592]	0.463 [0.318–0.614]	0.302 [0.253–0.354]
-an inactive class		0.519 [0.408–0.630]	0.537 [0.367–0.682]	0.698 [0.644–0.749]

Sensitivity (Se)	8	64.10 [48.47–77.70]	84.21 [63.16–95.05]	42.11 [32.54–52.15]

False-negative rate (under-classification, FNR)	9			
		35.90 [22.30–45.51]	15.79 [4.95–36.84]	57.89 [47.85–67.46]

Specificity (Sp)	10	73.81 [59.20–85.15]	63.64 [42.87–81.04]	85.91 [80.80–89.98]

False-positive rate (over-classification, FPR)	11			
		26.19 [14.86–40.80]	36.36 [0.1896–0.5712]	14.09 [10.02–19.20]

Positive predictivity (PP)	12	69.44 [53.32–82.51]	66.67 [46.76–82.76]	56.34 [44.74–67.43]

Negative predictivity (NP)	13	68.89 [54.49–80.89]	82.35 [59.63–97.48]	77.46 [72.59–81.80]

Post-test probability of classification	14			
-as active (PCA)		0.444 [0.340–0.553]	0.585 [0.433–0.726]	0.225 [0.177–0.281]
-as inactive (PCIC)		0.556 [0.447–0.660]	0.415 [0.274–0.567]	0.775 [0.7259–0.818]

Probability of a wrong classification	15			
-as active compound (PWCA)		0.306 [0.175–0.467]	0.333 [0.172–0.532]	0.437 [0.326–0.553]
-as inactive compound (PWCI)		0.311 [0.191–0.455]	0.177 [0.055–0.404]	0.225 [0.177–0.281]

Odds Ratio (OR)	16	5.03 [1.96–13.12]	9.33 [2.18–40.07]	4.43 [2.53–7.76]

**Table 2:** Diagnostic of classification model presented in Equation 2.

Parameter (Abbreviation)	Equation	Training (*n* = 88)	Test (*n* = 28)	External (*n* = 92)

χ^2^ statistic (*p*-value)		30.91 (*p* < 0.0001)	9.82 (0.0017)	28.76 (*p* < 0.0001)

Φ		0.5927	0.5922	0.5591

Accuracy (AC)	5	80.68 [71.45–87.80]	92.86 [78.12–98.17]	79.35 [70.19–86.58]

Error Rate (ER)	6	19.32	7.14	20.65

Prior proportional probability of	7			
-an active class		0.511 [0.408–0.614]	0.179 [0.074–0.350]	0.435 [0.337–0.537]
-an inactive class		0.489 [0.375–0.602]	0.821 [0.644–0.927]	0.565 [0.457–0.674]

Sensitivity (Se)	8	77.78 [64.06–87.87]	60.00 [20.97–90.51]	77.50 [62.85–88.14]

False-negative rate (under-classification, FNR)	9			
		22.22 [12.13–35.94]	40.00 [9.49–79.03]	22.50 [11.86–37.15]

Specificity (Sp)	10	83.72 [70.48–92.25]	100.00 [87.79–100.00]	80.77 [68.45–89.55]

False-positive rate (over-classification, FPR)	11			
		16.28 [7.78–29.52]	0.00 [0.00–12.21]	19.23 [10.45–31.55]

Positive predictivity (PP)	12	83.33 [70.48–92.25]	100.00 [36.84–100.00]	75.61 [60.69–86.63]

Negative predictivity (NP)	13	78.26 [64.76–88.14]	92.00 [75.85–97.97]	82.35 [70.12–90.77]

Post-test probability of classification	14	0.477 [0.375–0.581]		
-as active (PCA)		0.523 [0.419–0.625]	0.107 [0.034–0.265]	0.446 [0.347–0.548]
-as inactive (PCIC)		0.477 [0.375–0.581]	0.893 [0.735–0.966]	0.554 [0.452–0.653]

Probability of a wrong classification	15			
-as active compound (PWCA)		0.167 [0.079–0.302]	0.000 [0.000–0.122]	0.244 [0.134–0.390]
-as inactive compound (PWCI)		0.217 [0.119–0.352]	0.080 [0.020–0.242]	0.177 [0.092–0.299]

Odds Ratio (OR)	16	18.00 [6.25–52.39]	n.a.	14.47 [5.32–39.99]

Φ = coefficient of correlation in 2 × 2 contingency table; *χ*^2^ = Chi-squared statistic.

**Table 3:** Parameters for the characterization of prediction.

Parameter (Abbreviation)	Formula	Definition	Equation

Concordance/Accuracy/Non-error Rate (CC/AC)	100 × (*TP* + *TN*)/n	Total fraction of compounds correctly classified	6

Error Rate (ER)	100 × (*FP* + *FN*)/*n* = 1 − *CC*	Total fraction of compounds misclassified	7

Prior proportional probability of a class (PPP)	n_i_/n	Fraction of compounds belonging to class *i*	8

Sensitivity (Se)	100 × *TP*/(*TP* + *FN*)	Percentage of BBB+ compounds correctly assigned to the active class	9

False-negative rate (under-classification, FNR)	100 × *FN*/(*TP* + *FN*) = 1 − *Se*	percentage of BBB+ compounds falsely assigned to the non-active class	10

Specificity (Sp)	100 × *TN*/(*TN* + *FP*)	Percentage of BBB– compounds correctly assigned to the negative class	11

False-positive rate (over-classification, FPR)	100 × *FP*/(*FP* + *TN*) = 1 − *Sp*	Percentage of BBB– compounds falsely assigned to be active	12

Positive predictivity (PP)	100 × *TP*/(*TP* + *FP*)	Percentage of compounds correctly assigned as active out of all active assigned compounds	13

Negative predictivity (NP)	100 × *TN*/(*TN* + *FN*)	Percentage of non-active out of non-active assigned compounds	14

Probability of classification			15
as active (PCA)	(*TP* + *FP*)/*n*	- Probability to classify a compound as active (true positive & false positive)	
as inactive (PCIC)	(*FN* + *TN*)/*n*	- Probability to classify a compound as inactive (true negative & false negative)	

Probability of a wrong classification			16
as active compound (PWCA)	*FP*/(*FP* + *TP*)	Probability of a false positive classification	
as inactive compound (PWCI)	*FN*/(*FN* + *TN*)	Probability of a false negative classification	

Odds Ratio (OR)	(*TP* × *TN*)/(*FP* × *FN*)	The odds of correct classification in the group of active compounds divided to the odds of a incorrect classification in the group of inactive compounds	17

*TP* = number of true positive (BBB+ compounds classified as active); *TN* = number of true negative (BBB– compounds classified as non-active); *n* = sample size; *FP* = false positive (BBB- compounds classified as active); *FN* = false negative (BBB+ compounds classified as non-active); *n*_i_ = number of compounds belonging to class *i; i* = 1, 2 (where 1 = active BBB compounds; 2 = inactive BBB compounds).

**Table 4:** Compounds on training and test sets.

No.	Name	ID	logBB	Set	Ref.
1	Cimetidine	CID: 2756	−1.42	2	[37]
2	Icotidine	CID: 72108	−2.00	1	[37]
3	Lupitidine	CID: 51671	−1.06	2	[8]
4	Clonidine	CID: 2803	0.11	1	[37]
5	Mepyramine	CID: 4992	0.49	1	[37]
6	Imipramine	CID: 3696	0.83	1	[37]
7	Ranitidine	CID: 5039	−1.23	2	[37]
8	Tiotidine	CID: 50287	−0.82	1	[37]
9	Zolantidine	CID: 91769	0.14	2	[37]
10	Butanone	CID: 6569	−0.08	2	[8]
11	Benzene	CID: 241	0.37	1	[8]
12	3-Methylpentane	CID: 7282	1.01	1	[8]
13	3-Methylhexane	CID: 11507	0.90	1	[8]
14	2-Propanol	CID: 3776	−0.15	1	[8]
15	2-Methylpropanol	CID: 6560	−0.17	1	[8]
16	2-Methylpentane	CID: 7892	0.97	2	[8]
17	2,2-Dimethylbutane	CID: 580244	1.04	2	[8]
18	1,1,1-Trichloroethane	CID: 6278	0.40	1	[37]
19	Diethyl ether	CID: 3283	0.00	2	[8]
20	Enflurane	CID: 3226	0.24	1	[8]
21	Ethanol	CID: 702	−0.16	2	[8]
22	Fluroxene	CID: 9844	0.13	1	[8]
23	Halothane	CID: 3562	0.35	1	[8]
24	Heptane	CID: 8900	0.81	1	[8]
25	Hexane	CID: 8058	0.80	2	[8]
26	Isoflurane	CID: 3763	0.42	2	[8]
27	Methylcyclopentane	CID: 7296	0.93	2	[8]
28	Nitrogen	CID: 947	0.03	1	[8]
29	Pentane	CID: 8003	0.76	2	[8]
30	n-Propanol	CID: 1031	−0.16	2	[8]
31	Propanone	CID: 180	−0.15	2	[8]
32	Teflurane	CID: 31300	0.27	1	[8]
33	Toluene	CID: 1140	0.37	1	[8]
34	Acetylsalicylic acid	CID: 2244	−0.50	1	[8]
35	Pentobarbital	CID: 4737	0.12	1	[8]
36	Physostigmine	CID: 5983	0.08	2	[8]
37	Salicylic acid	CID: 338	−1.10	1	[8]
38	Trifluoro Perazine	CID: 5566	1.44	1	[8]
39	Valproic acid	CID: 3121	−0.22	1	[8]
40	Verapamil	CID: 2520	−0.70	1	[8]
41	Zidovudine	CID: 5726	−0.72	1	[8]
42	Hydroxyzine	CID: 3658	0.39	2	[8]
43	Thioridazine	CID: 5452	0.24	1	[8]
44	Alprazolam	CID: 2118	0.04	2	[8]
45	Phenserine	CID: 192706	1.00	1	[8]
46	Midazolam	CID: 4192	0.36	2	[8]
47	Codeine	CID: 5284371	0.55	2	[8]
48	Chlorpromazine	CID: 2726	1.06	2	[8]
49	Promazine	CID: 4926	1.23	1	[8]
50	Nevirapine	CID: 4463	0.00	1	[8]
51	Thioperamide	CID: 3035905	−0.16	1	[8]
52	Didanosine	CID: 3043	−1.30	2	[8]
53	Ibuprofen	CID: 3672	−0.18	1	[8]
54	Antipyrine	CID: 2206	−2.00	2	[38]
55	Theophyline	CID: 2153	−0.29	1	[8]
56	p-Acetamido phenol	CID: 1983	−0.31	1	[8]
57	Nitrous Oxide	CID: 948	0.03	1	[8]
58	Carbon bisulphide	CID: 6348	0.60	1	[8]
59	Indomethacin	CID: 3715	−1.26	1	[8]
60	Indinavir	CID: 5362440	−0.75	1	[8]
61	Oxazepam	CID: 4616	0.61	1	[8]
62	Carbamazepine	CID: 2554	−0.14	2	[39]
63	Carbamazepine epoxide	CID: 2555	−0.35	1	[39]
64	Amitriptyline	CID: 2160	0.88	1	[8]
65	Desipramine	CID: 2995	1.00	1	[8]
66	Mianserin	CID: 4184	0.99	2	[8]
67	ORG 4428	CID: 166560	0.82	2	[8]
68	Mirtazapine	CID: 4205	0.53	1	[8]
69	Tibolone	CID: 21844	0.40	1	[8]
70	Domperidone	CID: 3151	−0.78	2	[8]
71	Risperidone	CID: 5073	−0.67	2	[8]
72	9-OH-Risperidone	CID: 475100	−0.02	1	[8]
73	Temelastine	CID: 55482	−1.88	2	[8]
74	BBCPD13	CSID: 14922095	−0.66	1	[37]
75	BBCPD15	CSID: 2992532	−0.18	1	[37]
76	BBCPD57	CSID: 10439135	−1.15	2	[37]
77	BBCPD58	CSID: 10442225	−1.54	1	[37]
78	BBCPD17	CSID: 10442293	−1.12	1	[37]
79	BBCPD20	CID: 9971484	−0.46	1	[37]
80	BBCPD21	CID: 10498206	−0.24	2	[37]
81	SB222200	CSID: 3167851	0.30	1	[8]
82	Y-G14	CSID: 2276	−0.30	1	[8]
83	Y-G15	CSID: 72747	−0.06	1	[37]
84	Caffeine	CID: 2519	−2.00	1	[38]
85	Chlorambucil	CID: 2708	−1.60	1	[38]
86	Glycine	CID: 750	−3.50	2	[38]
87	Morphine	CID: 5288826	−2.70	2	[38]
88	Phenylalanine	CID: 994	−1.30	2	[38]
89	Phenytoin	CID: 1775	−2.20	1	[38]
90	Propranolol	CID: 4946	−1.20	1	[38]
91	Taurocholic Acid	CID: 444349	−4.10	1	[38]
92	Trichloroethylene	CID: 6575	0.34	1	[37]
93	Carmustine	CID: 450682	−0.52	1	[39]
94	ORG34167	CSID: 8036856	0.00	1	[8]
95	BBCPD22	CSDI: 8620184	−0.02	1	[37]
96	BBCPD23	BBCPD23	0.69	2	[37]
97	BBCPD24	BBCPD24	0.44	1	[37]
98	BBCPD26	BBCPD26	0.22	2	[37]
99	1,1,1-Trifluoro-2-chloro ethane	CSID: 6168	0.08	1	[37]
100	T7	T7	0.85	1	[37]
101	BBCPD60	CSDI: 23218171	−0.73	1	[37]
102	BBCPD18	BBCPD18	−0.27	1	[37]
103	BBCPD19	BBCPD19	−0.28	2	[37]
104	BBCPD16	BBCPD16	−1.57	1	[37]
105	BBCPD14	BBCPD14	−0.12	2	[37]
106	Y-G16	Y-G16	−0.42	1	[8]
107	Y-G19	Y-G19	−1.30	2	[8]
108	Y-G20	CSID: 5854406	−1.40	1	[37]
109	SKF89124	CSID: 117961.	−0.43	1	[8]
110	SKF101468	CSID: 4916	0.25	1	[37]
111	CBZ-EPO	CBZ-EPO	−0.34	1	[37]
112	L-663581	CSID: 114837	−0.30	1	[8]
113	M1L-663,581	CSID: 8560187	−1.34	1	[37]
114	M2L-663581	CSID: 8267285	−1.82	1	[8]
115	ORG5222	ORG5223	1.03	2	[8]
116	ORG12962	CSID: 7972174	1.64	1	[8]
117	ORG13011	ORG13011	0.16	1	[8]
118	ORG32104	ORG32104	0.52	1	[8]
119	ORG30526	ORG30526	0.39	1	[8]
120	ICI17148	ICI17149	−0.04	2	[37]
121	SK&F93319	SK&F93320	−1.30	1	[37]
122	CBZ	CBZ	0.00	1	[37]

CID = ID of compounds taken from PubChem; CSID = ID of compounds taken from ChemSpider.

**Table 5:** Summary statistical characteristics of training and test sets.

Parameter	Training set (*n* = 81)	Test set (*n* = 41)
m [95%CI]	−0.2003 [−0.4060; −0.0055]	−0.2529 [−0.5916; 0.0858]
StDev	0.9306	1.0731
Min	−4.10	−3.50
Max	1.64	1.06
KS statistic (p)	0.1151 (0.2163)	0.1729 (0.1531)
AD statistic *	1.1582 *	0.9939 *
CS statistic (p)	8.1850 (0.2249)	0.3650 (0.9852)

*m* = arithmetic mean; 95%CI = 95% confidence interval; StDev = standard deviation; *n* = sample size; KS = Kolmogorov-Smirnow test of goodness-of-fit; AD = Anderson-Darling test of goodness-of-fit; CS = Chi-Squared test of goodness-of-fit;

* critical value = 2.5018.

## References

[1] Rubin LL, Staddon JM (1999). The cell biology of the blood-brain barrier. Annu. Rev. Neurosci.

[2] Abraham MH, Ibrahim A, Zhao Y, Acree WE (2006). A data base for partition of volatile organic compounds and drugs from blood/plasma/serum to brain, and an LFER analysis of the data. J. Pharm. Sci.

[3] Klon AE (2009). Computational models for central nervous system penetration. Curr. Comput.-Aided Drug Des.

[4] Bechtold E, Reisz JA, Klomsiri C, Tsang AW, Wright MW, Poole LB, Furdui CM, King SB (2010). Water-soluble triarylphosphines as biomarkers for protein s-nitrosation. ACS Chem. Biol.

[5] Clark DE (2003). *In silico* prediction of blood-brain barrier permeation. Drug Discov. Today.

[6] Young RC, Mitchell RC, Brown TH, Ganellin CR, Griffiths R, Jones M, Rana KK, Saunders D, Smith IR, Sore NE (1988). Development of a new physicochemical model for brain penetration and its application to the design of centrally acting H2 receptor histamine antagonists. J. Med. Chem.

[7] Crivori P, Cruciani G, Carrupt PA, Testa B (2000). Predicting blood-brain barrier permeation from three-dimensional molecular structure. J. Med. Chem.

[8] Narayanan R, Gunturi SB (2005). *In silico* ADME modelling: prediction models for blood-brain barrier permeation using a systematic variable selection method. Bioorg. Med. Chem.

[9] Subramanian G, Kitchen DB (2003). Computational models to predict blood-brain barrier permeation and CNS activity. J. Comput.-Aided Mol. Des.

[10] Goodwin JT, Clark DE (2005). *In silico* predictions of blood-brain barrier penetration: Considerations to “keep in mind”. J. Pharmacol. Exp. Ther.

[11] Semple G, Andersson BM, Chhajlani V, Georgsson J, Johansson MJ, Rosenquist A, Swanson L (2003). Synthesis and biological activity of kappa opioid receptor agonists. Part 2: preparation of 3-aryl-2-pyridone analogues generated by solutionand solid-phase parallel synthesis methods. Bioorg. Med. Chem. Lett.

[12] Perioli L, Ambrogi V, Bernardini C, Grandolini G, Ricci M, Giovagnoli S, Rossi C (2004). Potential prodrugs of non-steroidal anti-inflammatory agents for targeted drug delivery to the CNS. Eur. J. Med. Chem.

[13] Hodgetts KJ, Yoon T, Huang J, Gulianello M, Kieltyka A, Primus R, Brodbeck R, De Lombaert S, Doller D (2003). 2-Aryl-3,6-dialkyl-5-dialkylaminopyrimidin-4-ones as novel crf-1 receptor antagonists. Bioorg. Med. Chem. Lett.

[14] Zhang H, Hu S, Zhang Y (2008). Prediction of distribution of neutral, acidic and basic structurally diverse compounds between blood and brain by the nonlinear methodology. Med. Chem.

[15] Klon AE (2009). Computational Models for Central Nervous System Penetration. Curr. Comput.-Aided Drug Des.

[16] Fan Y, Unwalla R, Denny RA, Di L, Kerns EH, Diller DJ, Humblet C (2010). Isights for predicting blood-brain barrier penetration of CNS targeted molecules using QSPR approaches. J. Chem. Inf. Model.

[17] Lanevskij K, Dapkunas J, Juska L, Japertas P, Didziapetris R (2011). QSAR analysis of blood-brain distribution: The influence of plasma and brain tissue binding. J. Pharm. Sci.

[18] Smye SW, Clayton RH (2002). Mathematical modelling for the new millenium: Medicine by numbers. Med. Eng. Phys.

[19] Sarbu C (1995). A comparative-study of regression concerning weighted least-squares methods. Anal. Lett.

[20] Okuno Y (2008). *In silico* drug discovery based on the integration of bioinformatics and chemoinformatics. Yakugaku Zasshi-J. Pharm. Soc. Jpn.

[21] Gozalbes R, Carbajo RJ, Pineda-Lucena A (2010). Contributions of computational chemistry and biophysical techniques to fragment-based drug discovery. Curr. Med. Chem.

[22] Loving K, Alberts I, Sherman W (2010). Computational approaches for fragment-based and *de novo* design. Curr. Top. Med. Chem.

[23] Sun H, Scott DO (2010). Structure-based drug metabolism predictions for drug design. Chem. Biol. Drug Des.

[24] Taherpour A (2010). Theoretical and quantitative structural relationship studies of electrochemical properties of the nanostructures of cis-unsaturated thiocrown ethers and their supramolecular complexes [X-UT-Y][M@C82] (M = Ce, Gd). Phosphorus, Sulfur Silicon Relat. Elem.

[25] Taherpour AA, Taherpour A, Taherpour Z, Taherpour O (2009). Relationship study of octanol-water partitioning coefficients and total biodegradation of linear simple conjugated polyene and carotene compounds by use of the Randic index and maximum UV wavelength. Phys. Chem. Liq.

[26] Hawkins DM (2004). The problem of overfitting. J. Chem. Inf. Comput. Sci.

[27] Durbin J, Watson GS (1950). Testing for serial correlation in least squares regression, I. Biometrika.

[28] Durbin J, Watson GS (1951). Testing for serial correlation in least squares regression, II. Biometrika.

[29] Picard R, Cook D (1984). Cross-validation of regression models. J. Am. Stat. Assoc.

[30] Kortagere S, Chekmarev D, Welsh WJ, Ekins S (2008). New predictive models for blood-brain barrier permeability of drug-like molecules. Pharm. Res.

[31] Cooper JA, Saracci R, Cole P (1979). Describing the validity of carcinogen screening tests. Br. J. Cancer.

[32] Bolboacă S, Jäntschi L, Achimaş Cadariu A (2004). Creating diagnostic critical appraised topics. catrom original software for romanian physicians. Appl. Med. Inf.

[33] Drugan T, Bolboacă S, Jäntschi L, Achimaş Cadariu A (2003). Binomial distribution sample confidence intervals estimation 1. sampling and medical key parameters calculation. Leonardo Electron. J. Pract. Technol.

[34] Bolboacă S, Jäntschi L (2008). Optimized confidence intervals for binomial distributed samples. Int. J. Pure Appl. Math.

[35] Jäntschi L, Bolboacă SD (2010). Exact probabilities and confidence limits for binomial samples: Applied to the difference between two proportions. The Scientific World JOURNAL.

[36] Steiger JH (1980). Tests for comparing elements of a correlation matrix. Psychol. Bull.

[37] Iyer M, Mishru R, Han Y, Hopfinger AJ (2002). Predicting blood-brain barrier partitioning of organic molecules using membrane-interaction QSAR analysis. Pharm. Res.

[38] Liu X, Tu M, Kelly RS, Chen C, Smith BJ (2004). Development of a computational approach to predict blood-brain barrier permeability. Drug Metab. Dispos.

[39] Rose K, Hall LH, Hall LM, Kier LB Modeling blood-brain barrier partitioning using topological structure descriptors.

[40] Bolboacă SD, Jäntschi L (2010). Computer assisted geometry optimization for *in silico* modeling. Comput Methods Progr Biomed.

[41] Bolboacă SD, Jäntschi L (2009). Comparison of quantitative structure-activity relationship model performances on carboquinone derivatives. The Scientific World JOURNAL.

[42] Bolboacă SD, Jäntschi L (2008). Modelling the property of compounds from structure: statistical methods for models validation. Environ. Chem. Lett.

